# Supplementary material for: A high-throughput microsphere-based immunoassay of anti-SARS-CoV-2 IgM testing for COVID-19 diagnostics
Source: PLoS One. 2021 Sep 2;16(9):e0248444. doi: 10.1371/journal.pone.0248444 (PMC8412265; doi:10.1371/journal.pone.0248444)
Supplement: Attachment — Submitted filename: reviewers questions and answers.docx [file pone.0248444.s001.docx]

PONE-D-21-09355
A High-throughput Microsphere-based Immunoassay of Anti-SARS-CoV-2 IgM Testing for COVID-19 Diagnostics
PLOS ONE

Dear Dr. Sha,

Thank you for submitting your manuscript to PLOS ONE. After careful consideration, we feel that it has merit but does not fully meet PLOS ONE’s publication criteria as it currently stands. Therefore, we invite you to submit a revised version of the manuscript that addresses the points raised during the review process.

Please submit your revised manuscript by Jun 21 2021 11:59PM. If you will need more time than this to complete your revisions, please reply to this message or contact the journal office at plosone@plos.org. When you're ready to submit your revision, log on to https://www.editorialmanager.com/pone/ and select the 'Submissions Needing Revision' folder to locate your manuscript file.

Please include the following items when submitting your revised manuscript:

- A rebuttal letter that responds to each point raised by the academic editor and reviewer(s). You should upload this letter as a separate file labeled 'Response to Reviewers'.
- A marked-up copy of your manuscript that highlights changes made to the original version. You should upload this as a separate file labeled 'Revised Manuscript with Track Changes'.
- An unmarked version of your revised paper without tracked changes. You should upload this as a separate file labeled 'Manuscript'.

If you would like to make changes to your financial disclosure, please include your updated statement in your cover letter. Guidelines for resubmitting your figure files are available below the reviewer comments at the end of this letter.

If applicable, we recommend that you deposit your laboratory protocols in protocols.io to enhance the reproducibility of your results. Protocols.io assigns your protocol its own identifier (DOI) so that it can be cited independently in the future. For instructions see: [http://journals.plos.org/plosone/s/submission-guidelines#loc-laboratory-protocols](https://urldefense.proofpoint.com/v2/url?u=http-3A__journals.plos.org_plosone_s_submission-2Dguidelines-23loc-2Dlaboratory-2Dprotocols&d=DwMGaQ&c=euGZstcaTDllvimEN8b7jXrwqOf-v5A_CdpgnVfiiMM&r=akG7cqwreijQvBDlTvgRBE3LYYgtJHy7K_lKZL_hwXo&m=uRfMpuNAmp_jCN2dfMCJpDL_x5AqGNRBIw5w2FSxA0I&s=PlzRkyLclxIwyRmoo64rQMLW4XZeILcZSxJeO1xfSEA&e=). Additionally, PLOS ONE offers an option for publishing peer-reviewed Lab Protocol articles, which describe protocols hosted on protocols.io. Read more information on sharing protocols at [https://plos.org/protocols?utm_medium=editorial-email&utm_source=authorletters&utm_campaign=protocols](https://urldefense.proofpoint.com/v2/url?u=https-3A__plos.org_protocols-3Futm-5Fmedium-3Deditorial-2Demail-26utm-5Fsource-3Dauthorletters-26utm-5Fcampaign-3Dprotocols&d=DwMGaQ&c=euGZstcaTDllvimEN8b7jXrwqOf-v5A_CdpgnVfiiMM&r=akG7cqwreijQvBDlTvgRBE3LYYgtJHy7K_lKZL_hwXo&m=uRfMpuNAmp_jCN2dfMCJpDL_x5AqGNRBIw5w2FSxA0I&s=j_X_UlStpBh07f1h6BLnNU818DM1c62R0o7go3ZeCPg&e=).

We look forward to receiving your revised manuscript.

Kind regards,

Chandrabose Selvaraj, Ph.D.
Academic Editor
PLOS ONE

Journal Requirements:

When submitting your revision, we need you to address these additional requirements.

1. Please ensure that your manuscript meets PLOS ONE's style requirements, including those for file naming. The PLOS ONE style templates can be found at

https://journals.plos.org/plosone/s/file?id=wjVg/PLOSOne_formatting_sample_main_body.pdf and

[https://journals.plos.org/plosone/s/file?id=ba62/PLOSOne_formatting_sample_title_authors_affiliations.pdf](about:blank)

2. We note that you have included the phrase “data not shown” in your manuscript. Unfortunately, this does not meet our data sharing requirements. PLOS does not permit references to inaccessible data. We require that authors provide all relevant data within the paper, Supporting Information files, or in an acceptable, public repository. Please add a citation to support this phrase or upload the data that corresponds with these findings to a stable repository (such as Figshare or Dryad) and provide and URLs, DOIs, or accession numbers that may be used to access these data. Or, if the data are not a core part of the research being presented in your study, we ask that you remove the phrase that refers to these data.

 	The data is now presented in Table 2.

3. Thank you for stating the following in the Competing Interests section:

The authors have declared that no competing interests exist

We note that one or more of the authors are employed by a commercial company: DiaCarta Inc

1.            Please provide an amended Funding Statement declaring this commercial affiliation, as well as a statement regarding the Role of Funders in your study. If the funding organization did not play a role in the study design, data collection and analysis, decision to publish, or preparation of the manuscript and only provided financial support in the form of authors' salaries and/or research materials, please review your statements relating to the author contributions, and ensure you have specifically and accurately indicated the role(s) that these authors had in your study. You can update author roles in the Author Contributions section of the online submission form.

Please also include the following statement within your amended Funding Statement.

“The funder provided support in the form of salaries for authors [insert relevant initials], but did not have any additional role in the study design, data collection and analysis, decision to publish, or preparation of the manuscript. The specific roles of these authors are articulated in the ‘author contributions’ section.”

If your commercial affiliation did play a role in your study, please state and explain this role within your updated Funding Statement.

We have included this in our cover letter.

2. Please also provide an updated Competing Interests Statement declaring this commercial affiliation along with any other relevant declarations relating to employment, consultancy, patents, products in development, or marketed products, etc.

Within your Competing Interests Statement, please confirm that this commercial affiliation does not alter your adherence to all PLOS ONE policies on sharing data and materials by including the following statement: "This does not alter our adherence to  PLOS ONE policies on sharing data and materials.” (as detailed online in our guide for authors http://journals.plos.org/plosone/s/competing-interests) . If this adherence statement is not accurate and  there are restrictions on sharing of data and/or materials, please state these. Please note that we cannot proceed with consideration of your article until this information has been declared.

Please include both an updated Funding Statement and Competing Interests Statement in your cover letter. We will change the online submission form on your behalf.

We have included this in our cover letter.

Please know it is PLOS ONE policy for corresponding authors to declare, on behalf of all authors, all potential competing interests for the purposes of transparency. PLOS defines a competing interest as anything that interferes with, or could reasonably be perceived as interfering with, the full and objective presentation, peer review, editorial decision-making, or publication of research or non-research articles submitted to one of the journals. Competing interests can be financial or non-financial, professional, or personal. Competing interests can arise in relationship to an organization or another person. Please follow this link to our website for more details on competing interests: http://journals.plos.org/plosone/s/competing-interests

Additional Editor Comments:

Now I have received the reviewer’s review reports and all three reviewers have recommended the manuscript for a substantial revision. Please review the manuscript in accordance with the reviewer's comments and submit, along with reference checks.


[Note: HTML markup is below. Please do not edit.]

Reviewers' comments:

Reviewer's Responses to Questions

**Comments to the Author**

1. Is the manuscript technically sound, and do the data support the conclusions?

The manuscript must describe a technically sound piece of scientific research with data that supports the conclusions. Experiments must have been conducted rigorously, with appropriate controls, replication, and sample sizes. The conclusions must be drawn appropriately based on the data presented.

Reviewer #1: Partly

Reviewer #2: Yes

Reviewer #3: Partly

2. Has the statistical analysis been performed appropriately and rigorously?

Reviewer #1: Yes

Reviewer #2: Yes

Reviewer #3: Yes

3. Have the authors made all data underlying the findings in their manuscript fully available?

The [PLOS Data policy](https://urldefense.proofpoint.com/v2/url?u=http-3A__www.plosone.org_static_policies.action-23sharing&d=DwMGaQ&c=euGZstcaTDllvimEN8b7jXrwqOf-v5A_CdpgnVfiiMM&r=akG7cqwreijQvBDlTvgRBE3LYYgtJHy7K_lKZL_hwXo&m=uRfMpuNAmp_jCN2dfMCJpDL_x5AqGNRBIw5w2FSxA0I&s=i5UikFb6a7v1W6a6dEO92YYCmObhePq9Ya3jyeCJumQ&e=) requires authors to make all data underlying the findings described in their manuscript fully available without restriction, with rare exception (please refer to the Data Availability Statement in the manuscript PDF file). The data should be provided as part of the manuscript or its supporting information, or deposited to a public repository. For example, in addition to summary statistics, the data points behind means, medians and variance measures should be available. If there are restrictions on publicly sharing data—e.g. participant privacy or use of data from a third party—those must be specified.

Reviewer #1: No

Reviewer #2: Yes

Reviewer #3: No

4. Is the manuscript presented in an intelligible fashion and written in standard English?

PLOS ONE does not copyedit accepted manuscripts, so the language in submitted articles must be clear, correct, and unambiguous. Any typographical or grammatical errors should be corrected at revision, so please note any specific errors here.

Reviewer #1: Yes

Reviewer #2: Yes

Reviewer #3: Yes

5. Review Comments to the Author

Please use the space provided to explain your answers to the questions above. You may also include additional comments for the author, including concerns about dual publication, research ethics, or publication ethics. (Please upload your review as an attachment if it exceeds 20,000 characters)

Reviewer #1: This article is well written.
There are some missing informations and discussion.
Here are my suggestions:

2.3 Assay procedure
What are the two blank wells used to calculate the ratio? You should describe if it is a human sample, or a synthetic sample.

The blank wells consist of 3 µL of S1 RBD protein coated magnetic beads, 87 µL of BlockAid™ Blocking Solution, and 10 µL PBS-1% BSA. This information was added to the manuscript’s Methods *2.3* *Assay procedure.*

.

Cut-off value is set to 45. You should indicate how this cut-off has been determined (same comment in the clinical performance section).

The cut-off value was calculated based the ratio distribution of 162 COVID-19 negative samples tested. This information was added to the manuscript ‘s Methods *2.3* *Assay procedure.*

.

3.1 Clinical Performance
Table 1: please replace “IgG Positive” / “IgG Negative” by “IgM Positive” / ”IgM Negative”

Have replaced. Thank you.

Healthy donors data should be presented and discussed.
Data ROC curve and AUC analysis should be performed. The methodology used to determine the Cut-off value should be presented.

According to your suggestion, the ROC curve is drafted and shown in Figure 2. The Area under the ROC Curves (AUCs) are 0.85, 0.67, and 0.38 for samples collected on 0-7 days, 8-14 days, and 2-8 weeks from symptom onset, respectively. This information was added to the manuscript.

Instead of “data not shown”, data generated with matched serum and EDTA plasma should presented to demonstrate that EDTA plasma can be used.

According to your suggestion, the data is now presented in Table 2.

3.3 Precision

“Inter-assay reproducibility (between-run precision) was evaluated by testing negative sample and positive sample on five separate runs “
Please correct: “Inter-assay reproducibility (between-run precision) was evaluated by testing one negative sample and two positive samples on five separate runs “

Have corrected. Thank you.

You should indicate in table 4 that ratio are presented. It would be interesting to give additional characterization of serum#36 and Serum#32 (for instance the level positivity on a reference test).

Table 4 (now Table 5) now indicated that ratios are presented.

I would suggest to mention the stability of the developed reagents.
we have tested the reagent stability and confirmed the shelf life of the kit is about 12 months.

4. Discussion
You should address a major question:
Why do you use Luminex technology in a simplex mode ?

You might want to present some preliminary data of an IgM and IgG kit.
Great question. We do have another kit for anti-SARS-CoV-2 IgG detection. Luminex Magpix is used in a simplex mode due to specific customer needs.

As a positive IgG test indicates protection against infection (i.e., sought after by people who are vaccinated) and a positive IgM test indicates recent infection (i.e., required by travelers flying to China), these two tests were rarely purchased together by our customers.

Reviewer #2: In this study, a high-throughput immunoassay of anti-SARS-CoV-2 IgM antibody was carried out on Luminex Magpix platform. A total of 42 serum samples from patients with positive COVID-19 were collected. This study has certain value for the control of SARS coronavirus. However, the authors should declare the creativity of this work and compare it in depth with other standard IgM tests。

This is a microsphere-based immunoassay. It is semi-quantitative assay and can give a quantitative IgM (MFI) for each patient. This is particle important when we discuss this with vaccinated individual. We have demonstrated infected individual has same amount IgM for N protein and S1 protein. However, for recovered and vaccinated individual, anti SARS-CoV-2 N IgM and S1 IgM has different amount which it can be distinguished each other (our different manuscript). It can run 96 samples each time during 3 hrs. It is quite different to LFI in the throughput and accuracy.

Reviewer #3: The anti-SARS CoV2 IgM assay described is rather straight forward one. The investigators have used commercially available recombinant SARS CoV2 Spike protein 1 (RBD) and conjugated with commercially available microsphere and all reagents used in the assay were commercial ones. Some of the points that need clarification are:

1. What was the source of Covid positive and covid negative patient sample? How as Covid positivity or negativity determined? This information is crucial since all conclusions on performance are based on these samples. Were the patients RT-PCR positive or seropositive? If latter, what was the methodology used and the status of seropositivity (igM or IgG or both?).

These samples were tested by FDA EUA IgM assay DIAZYME SARS-CoV-2 IgG/IgM CLIA kit and the patient were also confirmed by FDA EUA qPCR. We noted in section *2.2 Reagents and patient samples* that COVID-19 patient serum samples were acquired from ProMedDx. We noted in section *3.1 Clinical Performance* that the patients are RT-PCR positive.

2. As per section 3.4, there was data on igG positivity and IgM negativity for 10 samples from another commercial kit. What about others (totally 42 SARS CoV2 positive used).

The patient samples were acquired based on RT-PCR result thus not all samples were tested for antibodies.

3. The study was designed to test for SARS CoV2 IgM, but Table 1 shows data for IgG. What about IgM?

Sorry, it was an error. The table was corrected. The results shown are IgM tests instead of IgG. Sorry for the confusion.

[NOTE: If reviewer comments were submitted as an attachment file, they will be attached to this email and accessible via the submission site. Please log into your account, locate the manuscript record, and check for the action link "View Attachments". If this link does not appear, there are no attachment files.]

While revising your submission, please upload your figure files to the Preflight Analysis and Conversion Engine (PACE) digital diagnostic tool, [https://pacev2.apexcovantage.com/](https://urldefense.proofpoint.com/v2/url?u=https-3A__pacev2.apexcovantage.com_&d=DwMGaQ&c=euGZstcaTDllvimEN8b7jXrwqOf-v5A_CdpgnVfiiMM&r=akG7cqwreijQvBDlTvgRBE3LYYgtJHy7K_lKZL_hwXo&m=uRfMpuNAmp_jCN2dfMCJpDL_x5AqGNRBIw5w2FSxA0I&s=jxskIjLDqaqdVxNrGbW-tP61dB-xgT6mpJXZZFdd7b8&e=). PACE helps ensure that figures meet PLOS requirements. To use PACE, you must first register as a user. Registration is free. Then, login and navigate to the UPLOAD tab, where you will find detailed instructions on how to use the tool. If you encounter any issues or have any questions when using PACE, please email PLOS at [figures@plos.org](mailto:figures@plos.org). Please note that Supporting Information files do not need this step.
